# Galectin-7 in Epithelial Homeostasis and Carcinomas

**DOI:** 10.3390/ijms18122760

**Published:** 2017-12-19

**Authors:** Tamara Advedissian, Frédérique Deshayes, Mireille Viguier

**Affiliations:** Team Morphogenesis, Homeostasis and Pathologies, Institut Jacques Monod, UMR 7592 CNRS—University Paris Diderot, Sorbonne Paris Cité, 15 rue Hélène Brion, 75013 Paris, France; tamara.advedissian@ijm.fr (T.A.); frederique.deshayes@ijm.fr (F.D.)

**Keywords:** galectins, galectin-7, epithelia, carcinoma

## Abstract

Galectins are small unglycosylated soluble lectins distributed both inside and outside the cells. They share a conserved domain for the recognition of carbohydrates (CRD). Although galectins have a common affinity for β-galatosides, they exhibit different binding preferences for complex glycans. First described twenty years ago, galectin-7 is a prototypic galectin, with a single CRD, able to form divalent homodimers. This lectin, which is mainly expressed in stratified epithelia, has been described in epithelial tissues as being involved in apoptotic responses, in proliferation and differentiation but also in cell adhesion and migration. Most members of the galectins family have been associated with cancer biology. One of the main functions of galectins in cancer is their immunomodulating potential and anti-angiogenic activity. Indeed, galectin-1 and -3, are already targeted in clinical trials. Another relevant function of galectins in tumour progression is their ability to regulate cell migration and cell adhesion. Among these galectins, galectin-7 is abnormally expressed in various cancers, most prominently in carcinomas, and is involved in cancer progression and metastasis but its precise functions in tumour biology remain poorly understood. In this issue, we will focus on the physiological functions of galectin-7 in epithelia and present the alterations of galectin-7 expression in carcinomas with the aim to describe its possible functions in tumour progression.

## 1. Introduction

Galectins are a family of soluble lectins, which possess a large variety of ligands and functions. Among the galectins, galectin-7 presents a unique tissue-specific expression pattern and participates in diverse biological processes, notably in the regulation of epithelial homeostasis. In this review, after discussing general information about the galectin family, we will present galectin-7 expression profile and structure. Then, we will focus on the role of galectin-7 in epithelial homeostasis, in cell adhesion and migration by presenting the results obtained both in animal models and cell lines. Finally, we will address the association of galectin-7 with carcinoma and its putative function in cancer progression.

### 1.1. Galectins

Galectins were identified in the 1970s [1,2] and formerly named S-type lectins, due to their solubility and the sulfhydryl-dependency of the first galectins discovered [3], but their nomenclature became systematic in 1994. Since then, they were ranked according to their order of discovery [4]. Several galectins are expressed in the same species with up to 16 galectins identified in mammals and 12 in humans [5,6,7]. Galectins are a family of proteins characterized by a common affinity for β-galactoside containing carbohydrates and an evolutionary conserved Carbohydrate Recognition Domain (CRD) [4]. The different galectins do not possess any signal peptide or any anchoring domain and are synthesized by the free polysomes in the cytosol [7]. The unique exception is galectin-3, which possess a NES (Nuclear Export Signal) [8] and a NLS (Nuclear Localization Signal)-like motif with similarities with the NLS of p53 and c-Myc [9]. The galectins are secreted by an unconventional pathway and thus can be localized in the extracellular compartment [10,11]. However, they are also found in the cytosol, in the nucleus, or even in the mitochondria [11,12].

Moreover, while some galectins such as galectin-1 and galectin-3 are widely expressed, other family members have a more restricted tissue localisation. Hence, galectin-2 expression is limited to digestive epithelia [13] and galectin-7 is preferentially expressed in stratified epithelia [14].

**Figure 1:**
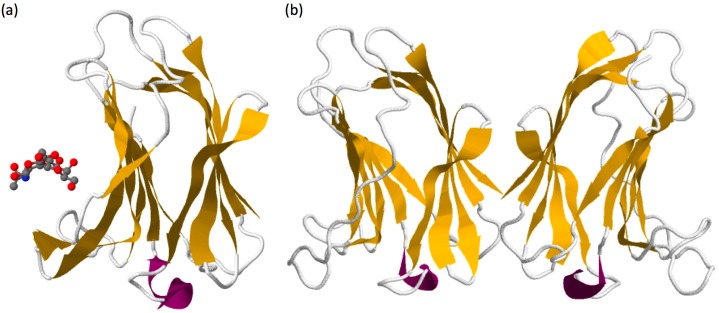
(**a**) Representation of the crystal structure of galectin-7 in complex with LacNAc (PBD 4XBQ) [30]. Carbohydrates are recognized by the residues H49, N51, R53, N62, W69, E72 and R74 forming the CRD. (**b**) Crystal structure of homodimeric galectin-7 (PBD 1BKZ) [18] illustrating the “back-to-back” arrangement of galectin-7 dimers with the two CRD orientated in the opposite direction. Structures obtained from www.rcsb.org.

Galectin sequences are similar from the lower invertebrates to mammals. The common basic structure of the galectin domain is composed of about 130 amino acids organised in two β-sheets containing five (F1–F5) and six (S1–S6) anti-parallel β-strands forming a jellyroll topology (see galectin-7 structure in Figure 1a) [4,15]. Seven carbohydrate-binding amino acids in strands S4, S5 and S6 are essential for the specific binding of β-galatosides [16] and are highly conserved among galectins [17,18]. These amino acids are encoded by three consecutive exons in mammalian galectins and form a characteristic sequence of galectins called the CRD [4].

According to the structural organization of their CRD, galectins can be classified into 3 subtypes. Hence, “proto-type” galectins, are composed of a single galectin domain that is able to dimerize (galectin-1, -2, -5, -7, -10, -11, -13, -14, and -15) whereas “tandem repeat-type” galectins possess a single polypeptide chain with two CRDs connected by a linker peptide (galectin-4, -6, -8, -9, and -12). The “chimera-type” subtype, with galectin-3 being the unique member, is constituted of one C-terminal CRD linked to a N-terminal non-lectinic domain [19,20].

Another classification of galectins, which is based on the determination of two CRD subtypes, also exists and refers to the evolution of galectins. The CRDs are thus defined according to the relative position of intron/exon corresponding to the sequence of the F4 or F3 β-sheet. Indeed, among the 3 exons encoding the CRD, there are two subtypes of the second exon, also called “W” exon because of the presence of a highly conserved tryptophan residue. One of the “W” exon ends within the sequence encoding the F4 β-strand and the other ends within the sequence encoding the F3 β-strand. These two subtypes have been called respectively F4-CRD and F3-CRD. Prototype galectins can belong to the F4-CRD subtype (e.g., galectin-7, galectin-10) or to the F3-CRD subtype (e.g., galectin-1, galectin-2) and galectin-3 contains a F3-CRD. The tandem-repeat galectins contain both a F4-CRD and a F3-CRD subtypes [7,20].

Thanks to their CRD, galectins recognize oligosaccharides present in proteins, lipids or microbial molecules. The minimal ligand recognized by galectins is *N*-Acetyl-Lactosamine (LacNAc), a disaccharide found on both *N*- and *O*-glycans [7,21]. However, galectins have a selective affinity for sugars with complex organisation according to their structure and composition: amount of branching, of LacNAc repeats (poly-LacNAc) or the presence of terminal saccharides such as sialic acid or fucose [21,22,23]. These differences enable the specific affinity of a given galectin for its ligand. In general, the affinity of galectins for complex carbohydrates increases with the number of LacNAc repeats and the number of branches. Hence the major ligands of galectins are *N*-glycans [21,24].

As a consequence, galectins can bind multiple glycosylated partners, either glycoproteins or glycolipids. Due to their multivalence, they form networks of molecules termed “lattice “ [25,26]. In addition to binding to glycans on glycoconjugates, galectins interact with unglycosylated intracellular but also extracellular ligands. As an illustration, galectin-1 has been shown to interact directly with the pseudo-light chain λ5 of the pre-BCR (B Cell Receptor) [27] and galectin-7 with Bcl-2 [12] or E-cadherin [28].

Due to their diversity of localisation and their various partners, the different members of the galectin family display a striking functional diversification. In particular, they are involved in intracellular trafficking as well as in cell adhesion and cell migration, in the regulation of the immune system or even in mRNA splicing [11]. Galectins can also affect cell signalling and impact development and tissue homeostasis leading to the emergence of pathologies such as cancer [7,25,29].

### 1.2. Galectin-7

The galectin-7 gene was simultaneously discovered by Madsen and colleagues [31] and Magnaldo and colleagues [32] to be expressed in the epidermis, respectively as a protein repressed during Simian Virus-40 (SV-40)-mediated transformation of keratinocytes and as a protein differentially expressed in normal keratinocytes and in squamous cancer cells which failed to complete terminal keratinocyte differentiation. This lectin is mostly expressed in stratified epithelia notably in the epidermis (where galectin-7 is found both in interfollicular region and in hair follicles), the oesophagus, the tongue, the anus, the lips and the cornea [14,33,34]. However, galectin-7 expression has also been described in thymic Hassall’s corpuscles [14], in sebaceous glands [33] and in myoepithelial cells from the mammary epithelia [35]. Galectin-7 has also recently been detected in the gingiva [36]. Importantly, its expression is induced by p53 and Ultra-Violet B (UVB) light [37]. Galectin-7 is secreted by keratinocytes into their culture medium despite the fact that, as all the galectins, it does not possess a typical secretion signal peptide [31]. However, galectin-7 is also found in the cytosol, in mitochondria and the nucleus, but its function in the nucleus is largely unknown. Diverse studies suggest an intracellular function of galectin-7 as for example in the regulation of keratinocyte proliferation and differentiation through the c-Jun N-terminal Kinase (JNK1)–miR-203-p63 pathway (see below) [38]. Galectin-7 expression can be induced via p53 or TNFα activation pathways, and both wild type and p53 mutants harbouring “hot spot” point mutation bind the galectin-7 promoter [39]. In addition, Nuclear Factor-kappa B (NF-κB) also binds to the galectin-7 promoter [39].

Galectin-7 is widely present in mammals and only one orthologue has been described outside of the mammalian lineage in anol lizards [40]. Interestingly, a copy-number variation has been pointed out for galectin-7 for which a single copy of two genes has been identified notably in the human, cow and dog genome [40]. The *LGALS7* and *LGALS7B* genes have been duplicated in tandem but in opposite direction and are found in chromosome 19 in humans [40]. Both genes encode identical galectin-7 protein but exhibit different putative transcription factors binding sites in their promoter sequence suggesting differences in expression regulation [40]. It has been hypothesized that galectin-7 could come from a duplication of galectin-4 [20] which is present in its neighbourhoods as a single copy.

Galectin-7, as other prototypic galectins, is able to form homodimers but with a different topology. Indeed, despite sequence homologies with other prototypic galectins such as galectin-1 or galectin-2 that associate in dimer in a “side-by-side” organisation, galectin-7 form homodimer through a “back-to-back” arrangement giving rise to a larger dimer interface compared to other prototypic galectins (Figure 1b) [18,41]. This difference in structural arrangement suggests that the glycoconjugate bridging activity of galectin-7 may differ from other prototypic galectins. The substitution at position 74 of an arginine by a serine inhibits the carbohydrates-binding activity of galectin-7 but does not alter the capacity of galectin-7 to form homodimers in solution [42]. This indicates that binding to oligosaccharides is not required for galectin-7 to form homodimers even if it can slightly modify its conformation and influence the dimers’ stability [43,44]. Regarding carbohydrate binding, galectin-7 displays preferential binding to internal or terminal LacNAc repeat carried by *N*-glycan (Figure 1a) [21,22].

**Figure 2:**
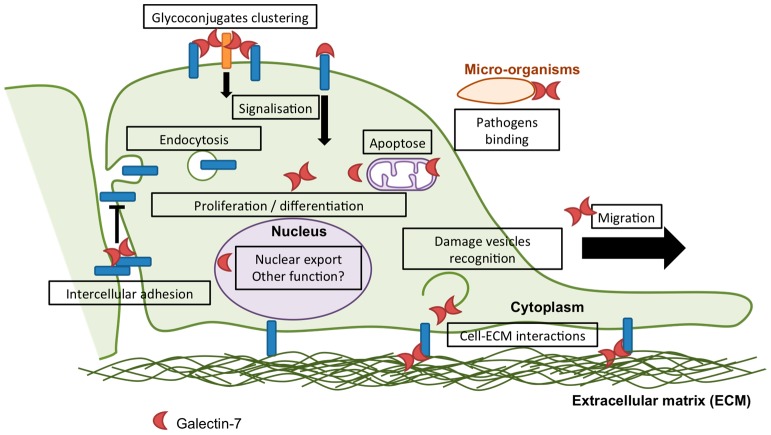
Schematic representation of known and putative functions of galectin-7. In addition to the functions of galectin-7 in cell proliferation, apoptosis, differentiation, migration and adhesion described in this issue, few evidence highlights other functions of galectin-7 in epithelia. As an illustration, galectin-7 has been shown to interfere with Transforming Growth factor β (TGFβ) signalling in response to Hepatocyte Growth Factor (HGF) by promoting smad3 export from the nucleus and thus preventing liver fibrosis occurrence [45]. In addition, the commensal bacteria *Finegoldia magna* has been described to adhere to the upper layers of the epidermis through binding of the adhesion bacterial protein *F. magna* Adhesion Factor (FAF) to galectin-7, indicating that galectin-7 can bind to ligands from microbial origin [46].

Multiple cellular functions have been attributed to galectin-7, most of which are related to epithelial integrity maintenance and will be described below. However, the established and putative cellular functions of galectin-7 are summarized in Figure 2.

## 2. Galectin-7 in Epithelial Homeostasis

Galectin-7 participates in epithelial maintenance by regulating at least three key aspects of epithelia homeostasis: cell growth, cell differentiation and apoptosis. Nevertheless, the precise mechanisms by which galectin-7 participate in the regulation of these processes still remain to be decrypted more deeply.

### 2.1. Apoptosis

Several studies have revealed a role of galectin-7 in the apoptotic response (Figure 2) [34]. However, depending on experimental conditions, galectin-7 has been shown to be either a pro-apoptotic factor or an anti-apoptotic factor, indicating that galectin-7 activity in apoptosis varies according to the cellular context and/or the apoptotic stimulus. First, it was discovered, in the epidermis, that UVB-induced sunburns increase galectin-7 expression in keratinocytes from human skin ex vivo [37]. Remarkably, overexpression of galectin-7 occurs in apoptotic keratinocytes, highlighting a possible association [37]. This has been demonstrated using mouse models lacking or overexpressing galectin-7 in the epidermis in which both absence and excess of galectin-7 modify the kinetics of the apoptosis response to UVB irradiation and induce premature apoptotic response [47,48], pointing out the involvement of galectin-7 in the apoptosis process in vivo.

Addition of recombinant galectin-7 in absence of apoptotic stimuli is sufficient to induce apoptosis in the T lymphocyte Jurkat cell line [41,49,50] and in freshly isolated human T cells [50], as previously shown for other galectins [51]. Apoptosis induction by galectin-7 in Jurkat cells can be inhibited by lactose addition, indicating that this function of galectin-7 relies on its lectin activity [50]. However, in other cell types, addition of recombinant galectin-7 [50] or alterations of galectin-7 expression levels alone [52,53] are not sufficient to induce apoptosis indicating that direct induction of apoptosis by galectin-7 is restricted to T lymphocytes.

To investigate the function of galectin-7 in apoptosis, most researchers induce the ectopic expression of galectin-7 in diverse cancer cell lines and examine the sensitivity of the cells to apoptotic stimuli. As an illustration, de novo expression of galectin-7 in the cervical cancer HeLa cells and in the colorectal adenocarcinoma DLD-1 cell line makes these cells more sensitive to the induction of apoptosis by UVB irradiation or diverse chemical apoptotic stimuli [12,54,55,56]. Similarly, overexpression of galectin-7 in ST88-14 cells, a sarcoma-derived cell line, in the cervical carcinoma siHa cells or in the prostate cancer cells DU-145 results in an increased susceptibility of the cells to apoptotic stimuli [56,57,58]. Accordingly, galectin-7 downregulation in the cervical squamous carcinoma cells SiHa and C-33A increases cell viability in response to the apoptosis-inducing chemotherapeutic agent paclitaxel [59]. All these studies indicate that galectin-7 has a pro-apoptotic effect in many cell types. However, in the breast MCF-7 cancer cells or in the B16F1 melanoma cell line, ectopic expression of galectin-7 decreases the cell sensitivity to apoptotic stimuli [42,60], indicating that galectin-7 can also, contrastingly, have an anti-apoptotic effect.

Interestingly, the function of galectin-7 in apoptosis does not rely on its lectin activity and is predominantly intracellular. Indeed, St-Pierre and colleagues have shown that the de novo expression of a CRD-defective galectin-7 mutant harbouring a substitution of an arginine by a serine at position 74 (R74S mutant) had a similar effect to the expression of the wild type galectin-7 on apoptosis susceptibility. These results were observed in both DU-145 prostate cancer cells where galectin-7 has a pro-apoptotic effect [58] and in MCF-7 breast cancer cells where galectin-7 had an anti-apoptotic function [42]. Nevertheless, how galectin-7 regulates apoptosis is still unclear. Kuwabara et al., reported that galectin-7-overexpressing cells exhibit increased cytochrome c release and amplified JNK activation after apoptosis stimulation indicating that galectin-7 acts upstream of these two pathways [54]. The mechanism by which galectin-7 participates in apoptosis could also be linked to its interaction with the anti-apoptotic factor Bcl-2. Indeed, galectin-7 directly interacts with Bcl-2 in a carbohydrate-independent manner [12]. In accordance, overexpression of galectin-7 increases cell apoptosis in response to a specific Bcl-2 inhibitor [56]. Remarkably, the R74S galectin-7 mutant, which localizes far less efficiently to the mitochondria, still contributes to apoptosis regulation [58], suggesting that galectin-7 may also function outside of the mitochondria.

### 2.2. Proliferation

Studies performed on diverse cell types, mostly cancerous cell lines, have demonstrated that galectin-7 has a suppressive effect on cell proliferation (Figure 1). Indeed, ectopic expression or addition of exogenous galectin-7 in the DLD-1 human colon carcinoma cell line [55] and the neuroblastoma cells SK-N-MC, respectively [53], drastically reduced tumour cell proliferation. Consistently, galectin-7 knockdown in human keratinocytes results in a hyperproliferative phenotype [38]. However, ectopic expression of galectin-7 in the B16F1 melanoma cell line did not affect cell growth [60] indicating that cell context might be important for galectin-7 to modulate cell proliferation. Evidence obtained in vivo in galectin-7-null mice indicates that galectin-7 is also involved in the regulation of cell growth during stress responses. Indeed, galectin-7 deficiency induces enhanced cell proliferation after epidermal injury or UVB irradiation of the skin [47].

The molecular mechanism by which galectin-7 participates in apoptosis and cell proliferation remain to be clarified. However, galectin-7 could be an effector of the tumour suppressor gene *p53*. Strikingly, galectin-7 expression is strongly induced by p53 [61] and lack of wild type p53 in human keratinocytes cell lines prevents galectin-7 expression induction in response to UVB irradiation [37].

### 2.3. Differentiation

Both proliferating basal and quiescent differentiated suprabasal keratinocytes express and secrete galectin-7 [14,31,32]. As a consequence, galectin-7 was described as a marker of stratified epithelia but not as a marker of differentiation. However, some evidence has suggested a role of galectin-7 in keratinocyte differentiation (Figure 2) such as its reduced expression after addition of retinoic acid in cultured keratinocytes [32]. In addition, in keratinocytes cultured in vitro, galectin-7 mRNA expression increases with the cell density, suggesting a potential link with epidermal differentiation [62]. Regarding tumour biology, galectin-7 downregulation correlates with poor tumour differentiation in bladder squamous cell carcinomas [63] and in vulvar squamous cell carcinoma [64]. Recently, Liu and colleagues produced the first mechanistic evidence of a function of galectin-7 in keratinocyte differentiation in vitro [38]. In fact, in the keratinocyte cell line HaCaT, galectin-7 knockdown reduces cell differentiation as assessed by the expression of keratins. Moreover, they found that intracellular galectin-7 regulates keratinocyte differentiation through the JNK–miR-203-p63 pathway [38]. Indeed, their results indicate that galectin-7 interacts with JNK1 and prevents its ubiquitination and subsequent degradation by the proteasome. Both galectin-7 and JNK1 induce miR-203 expression and the subsequent inhibition of the transcription factor p63, an important regulator of keratinocyte proliferation and differentiation [65].

## 3. Galectin-7 in Adhesion and Migration

In this section, we will describe current understanding of galectin-7 function in two interconnected mechanisms: cell migration and adhesion (Figure 2). Both processes are required for tissue maintenance and are central in tumour biology. As a consequence, modification of cell migration and adhesion characteristics can promote cancer progression.

### 3.1. Adhesion

Several galectins have been implicated in cell–cell or cell–ECM (Extra-Cellular Matrix) adhesion and thus, galectins are considered as a family of adhesion-modulating proteins [66]. However, depending on conditions (i.e., galectin considered, galectin concentration or cell types) galectins can either favour or prevent interactions with either the substrate or the neighbouring cells. Regarding galectin-7, its subcellular localisation is enriched at cell-cell contacts in the suprabasal layers of human and mouse epidermis [31,47]. However, the potential role of galectin-7 in intercellular adhesion is poorly documented. Recently, a few studies came out indicating that galectin-7 mediates cell-cell adhesion. Indeed, in the uterus, Menkhorst and colleagues showed that galectin-7 is expressed in the endometrium and influenced trophoblast-endometrial epithelia intercellular adhesion in vitro [67]. This function of galectin-7 in cell-cell adhesion may have a crucial impact during embryo implantation.

In addition, our team previously showed that galectin-7 interacts with the adherent junctions-component E-cadherin in keratinocytes [48]. Interestingly, we recently demonstrated that galectin-7 directly binds to the extracellular domain of E-cadherin in a glycosylation-independent manner [28]. This interaction has a functional consequence on intercellular adhesion as galectin-7 knockdown in HaCaT keratinocytes importantly reduces adherent junction-mediated adhesion [28]. Focusing on the underlying mechanisms, we demonstrated that galectin-7 stabilises E-cadherin at the plasma membrane, preventing its endocytosis [28]. Interestingly, both galectin-7-null mice and galectin-7-overexpressing mice show intercellular adhesion defects in the epidermis [48]. These results are compatible with the current model for the regulation of adhesion by galectins. In this model, low concentration of galectins will promote bridging of molecules by bi- or multivalent galectins and favour adhesion. On the contrary, high amount of galectins will reduce their crosslinking properties by decreasing the probability of simultaneous binding to two or more ligands and thus decreasing adhesion [66]. As a consequence, absence or excess of galectins could have the same consequences.

Regarding the regulation of the adhesion to the ECM, a possible interaction between galectin-7 and β1-integrin has been suggested in polarised MDCK cells [68]. Consistently, the enhanced endometrial wound repair induced by addition of exogenous galectin-7 is prevented by the blockade of integrin–fibronectin interaction in vitro [69]. However, further investigations are needed to specify the potential link between galectin-7 and integrins. Finally, galectin-7 could also participate in cell-ECM adhesion by influencing Matrix Metallo-Proteinase proteins (MMP) expression. Indeed, in lymphoma cells or in HeLa cells, exogenously added galectin-7 was able to enhance MMP-9 expression, suggesting a potential role for galectin-7 in the regulation of cell–ECM adhesion during cancer dissemination [70,71]. Galectin-7 and MMP-9 also showed positive expression correlation in human hypopharyngeal and laryngeal squamous cell carcinomas [72].

### 3.2. Migration

Galectin-7 has first been found to be involved in cell migration during epithelial wound healing in mouse corneas where addition of exogenous galectin-7 accelerated re-epithelialisation after corneal injury [73,74]. Simultaneous addition of lactose with exogenous galectin-7 prevented the increase of healing rate due to galectin-7 supplement [73,74], suggesting that this function of galectin-7 in corneal healing might be dependent on its binding to extracellular glycoconjugates. In mice corneas and porcine skin epidermis, galectin-7 expression is increased following injury [73,74,75,76], indicating that galectin-7 expression can be induced under stress conditions such as injury occurrence. Then, studies performed in galectin-7-null mice revealed that galectin-7 is similarly involved in keratinocyte migration during epidermal wound healing [47]. These mice displayed re-epithelialisation delay when compared to Wild Type (WT) mice after tail superficial scratch. This role of galectin-7 in epidermal wound healing is independent of its function in cell growth regulation but is related to a reduced migratory potential of keratinocytes. Indeed, the delay in wound healing was observed as soon as 24h h after injury, whereas no difference in cell proliferation was observed at this period in galectin-7-null mice compared to WT mice. In addition, in the presence of the cell division inhibitor mitomycin, the delay in wound healing was still observed in newborns’ skin explants from galectin-7-deficient mice compared to WT mice [47]. Surprisingly, overexpression of galectin-7 in mice epidermis also delayed wound closure after superficial epidermal injury [48], indicating that an optimal amount of galectin-7 is required for proper keratinocyte migration. Focusing on the underlying mechanisms, we have recently showed in vitro that galectin-7-depleted keratinocytes (HaCaT cells) have a reduced cell migration speed but also an impaired collective behaviour resulting in a decreased migration efficiency [28]. These alterations have been hypothesized to be related to the defective adherent junction functioning after galectin-7 silencing [28]. A role of galectin-7 in endometrial epithelial wound repair has also been highlighted by in vitro assays [69].

The function of galectin-7 in collective cell migration is relevant in pathological conditions such as cancer progression [34]. Indeed, in epithelial cancer, invasion processes of the surrounding healthy tissue by tumour cells frequently exhibit collective invasion reminiscent of regenerative migration of epithelial cells [77,78]. Accordingly, several groups reported an association between galectin-7 expression levels and cancer aggressiveness as we will discuss in the following section.

## 4. Galectin-7 and Carcinomas

Galectins are widely studied in cancer with several hundred references. Most members have been implicated as being associated either as markers, diagnostic cues, candidate effectors in cancer progression or modulators of treatment responses [29,79,80,81,82,83,84]. Interestingly, several galectins are usually co-expressed by a single cell or tumour and may exert ill-defined synergistic or compensatory properties.

### 4.1.Galectin-7 as a Tumour Progression Marker

Among these galectins, galectin-7, being expressed mostly in stratified epithelia, has been mainly studied in carcinomas and its implications in cancer were referenced only in a few tens of published articles. Among those, even fewer studies explored the ectopic expression of galectin-7 in tumours from non-epithelial origins such as lymphoid or melanomas tumours.

In lymphomas, ectopic expression of galectin-7 was shown to correlate with the metastatic potential of transplanted lymphomas cell lines [83,85,86,87]. Further observations in human lymphoid diseases suggested correlations between tumour progression and accumulation of galectin-7 [86] while no expression was detected in normal tissues.

Studies were also conducted to investigate the role of galectin-7 in melanoma. Indeed, tissue analysis on human melanomas and nevi first showed an expression of galectin-7 [88] but in situ labelling of nevi biopsies suggested that they were mostly the keratinocytes subpopulation of the biopsies that expressed galectin-7 [60]. In addition, it has been found that when the melanoma cells B16F1 are injected subcutaneously into mice, galectin-7 can be expressed by the resulting primary tumour as well as in lung metastasis [60]. However, even if galectin-7 increased the resistance of melanoma cells to apoptosis, studies on the melanoma cell line B16F1 showed that galectin-7 ectopic expression did not impact tumour growth and metastasis occurrence when cells are injected into mice [60].

Contrastingly, one of the major skin cancers apart from melanomas, basal cell carcinoma, appeared to be devoid of galectin-7 expression [89] while galectin-7 is expressed in normal tissues. Other carcinomas are also associated with decreased galectin-7 expression such as stomach [90], urothelial [91,92] and cervix [59] cancer. On the contrary, squamous epithelial and mucous tumours from head and neck [93], oesophagus [94] and thyroid [95] cancer do express higher galectin-7 levels than normal tissues.

In breast cancer, the earliest report on galectin-7 being associated to oncogenesis is from a chemically induced mammary tumour in rat which was found to overexpress galectin-7 contrastingly to chemically induced colon carcinomas which express a reduced level of galectin-7 [96]. Later on, galectin-7 was scored among the differentially expressed gene on human breast cancer [97]. Both galectin-3 and galectin-7 were associated with mammary tumour progression with galectin-7 being correlated to pejorative diagnosis when accumulated while reduced galectin-3 was of better prognosis [98]. Interestingly, these two galectins are mutually exclusive in mammary epithelia with galectin-7 being a marker of myoepithelial cells and luminal cells being galectin-3 positive [35]. The myoepithelial cells exhibit a basal type phenotype with a proliferative capacity [99,100]. Most importantly, galectin-7 is overexpressed in basal-like breast tumours [97,101]. Recently, using a mouse model for ErbB2 mammary tumours, galectin-7 expression was reported to accelerate tumour progression and the formation of tumour nodules in ErbB2-positive tumours [102]. On the contrary, using mouse model knocked-out for galectin-3 expression, no correlation was found with breast tumour progression or metastasis induced by transgenic expression of Mouse Mammary Tumour Virus- Polyoma Middle T (MMTV-PyMT) oncogene [103].

Furthermore, overexpression of galectin-7 was associated to metastatic potential of various tumour tissues, notably in breast cancer [101]. In oral squamous cell carcinoma (OSCC), galectin-7 has been associated with the histological malignancy grading system [104,105]. Consistently, in OSCC cell lines, galectin-7 downregulation reduces cell migration and invasion whereas its overexpression enhances cell migration and invasion of cancer cells [52]. Similarly to OSCC, in epithelial ovarian cancer, various studies described a correlation between galectin-7 ectopic expression in this monostratified epithelia and overall patient survival [50,106]. In addition, de novo galectin-7 expression was associated with progression to high-grade tumours and was enriched in metastatic samples compare to low-grade tumours [50]. On the contrary, in colon cancer, galectin-7 ectopic overexpression prevented metastatic dissemination [55] and promoted apoptosis after apoptosis induction by stimuli [54].

### 4.2. Galectin-7 as a Therapeutic Tool

The association of galectins with cancer [29,107] may be related to the well-known alterations in glycosylation signatures of cancer cells that may have incidental implications on galectins reactivity [108]. Indeed, during tumour progression, different factors affect protein glycosylation leading to altered glycan structure and composition as the appearance of truncated glycosylation motifs or enrichment of polyLacNAc [108,109,110]. This modification of galectin ligands will necessarily affect galectin activity and functions and can impact cell growth, adhesion, immunomodulation and cell migration [109]. As an illustration, modification of the surface glycome regulates galectin-1 binding to endothelial cells, and more importantly to VEGFR2, thus affecting angiogenesis and response to anti-VEGF treatments [111]. Among carcinomas, galectins-1 and -3 are the most studied and are already targeted in clinical trials [107,111,112]. Interestingly, immunomodulating potential is one of the most powerful actions of these galectins in cancer. Galectin-1 also has a relevant anti-angiogenic potential [113]. Another relevant function of galectins in cancer biology is cell migration and particularly adhesion to the MEC and to other cells (including surrounding or endothelial cells) [79]. As a consequence, galectin-7 appeared in the dedicated studies as a potential target for clinical approaches either as a prognostic marker, a direct modulating protein in cancer or a therapeutic target for inhibitors.

Indeed, galectin-7 has been proposed to serve as a marker in patients with certain type of cancer. Interestingly, galectin-7 overexpression on tumour tissues could be correlated to patient survival notably in ovarian [114] or in cervical [59,115] carcinomas. Furthermore, galectin-7 expression has been associated with better survival after chemo- or radio-therapies [91,115] and has been proposed to serve as a predictive marker of chemo- and radio-therapy resistance [116].

Administration of pure recombinant galectin-7 could be efficient in immunosuppressive approaches targeting T lymphocytes but contrastingly it could enhance invasive potential of tumour cells [50]. Soluble exogenously added galectin-7 has been used successfully to repair corneal injury in mice cornea ex vivo [73,74]. In a recent article, St-Pierre and colleagues demonstrated that recombinant galectin-7 added on tumour cells may rapidly traffic through intracellular compartments including endocytic vesicle and mitochondria and could induce ectopic expression of galectin-7 [117]. These new coming results reinforce the rationale of using galectin-7 on tumour that would benefit from its induced expression, as for example colon cancer [54,55]. On the contrary, using inhibitors of galectin-7 has to be tested in tumours where its expression is associated with pejorative prognosis.

**Figure 3:**
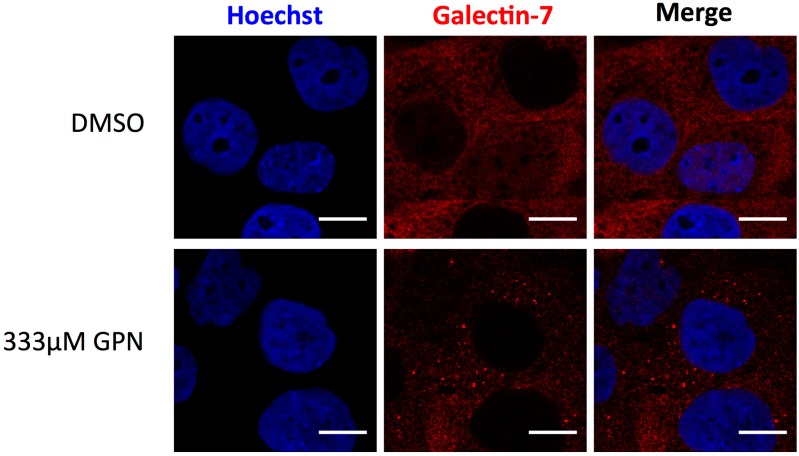
Galectin-7 is recruited at damaged lysosomes. Twelve minutes incubation with the lysosome-damaging agent GPN (glycyl-l-phenylalanine 2-naphthylamide) induces intracellular accumulation of galectin-7 at damaged lysosomes in HaCaT cells. Scale bar = 10 μm.

Moreover, development of specific galectin-7 inhibitors that will selectively target the intracellular or extracellular functions of galectin-7 could be a strategy to inhibit not all but specific galectin-7-mediated processes [118]. Interestingly, inhibition of the intracellular lectin-activity of galectin-7 could be easily assessed. As previously demonstrated for galectins-1, -3, -8 and -9 [119], galectin-7 is able to detect damages to vesicles (Figure 2 and Figure 3). Indeed, addition of a lysosomal-damaging agent induces intracellular recruitment of galectin-7 to permeabilized lysosomes indicating that this lectin recognizes altered vesicles (Figure 3). This feature shared by some galectins allows to identify galectin inhibitors that penetrate the cells and target the CRD domain of a given galectin, providing a functional assay to assess drug penetration and specificity [120,121]. Hence, some galectins are efficient to detect damaged vesicular compartment by binding to glycoproteins abnormally exposed in the cytoplasm due to vesicle integrity failure [119]. This approach is particularly relevant in the biomedical area where knowing the precise way of action of a chemical compound is a benefit.

## 5. Conclusions

Galectin-7 is a prototypic galectin, which is preferentially found in stratified epithelia where it favours epithelial homeostasis. Though galectin-7-null mice or mice overexpressing galectin-7 in the epidermis are viable and fertile, they present defective responses to stress conditions. Indeed, acting intra- or extracellularly, galectin-7 participates in diverse processes such as the susceptibility to apoptosis, cell migration and cell-adhesion. However, the detailed mechanism of action of this tissue-specific galectin and its partners are mostly unknown. Thus, further work is required to uncover galectin-7 functions and ways of action. This is particularly important regarding the biomedical field because galectin-7 could influence tumour progression. Hence, specifying how galectin-7 influences a given process could help to design and predict the effects of galectin-7 inhibitors targeting diverse regions of the protein or generated recombinant mutant galectin-7 that retain only some of the functions of galectin-7.

## Abbreviations

BCR	B-Cell Receptor
CRD	Carbohydrate Recognition Domain
DMSO	Dimethyl Sulfoxide
FAF	*F. magna* Adhesion Factor
ECM	Extracellular Matrix
HGF	Hepatocyte Growth Factor
JNK	c-Jun N-terminal Kinase
LacNAc	*N*-Acetyl-Lactosamine
MMP	Matrix Metalloproteinase
MMTV-PyMT	Mouse Mammary Tumor Virus – Polyoma Middle T
NES	Nuclear Export Signal
NF-κB	Nuclear Factor kappa B
NLS	Nuclear Localisation Signal
OSCC	Oral Squamous Cell Carcinoma
SV-40	Simian Virus-40
TGFβ	Transforming Growth Factor β
UVB	Ultraviolet B
WT	Wild Type
